# Beta cell glucose sensitivity identifies clinical response to disease-modifying therapies initiated at stage 3 type 1 diabetes onset

**DOI:** 10.1007/s00125-026-06703-8

**Published:** 2026-03-12

**Authors:** Carmella Evans-Molina, Stephen E. Gitelman, Andrea Mari, Ele Ferrannini

**Affiliations:** 1 Indiana University School of Medicine, Indianapolis, IN USA; 2 Center for Diabetes and Metabolic Diseases, Indiana University School of Medicine, Indianapolis, IN USA; 3 Department of Pediatrics and the Herman B Wells Center for Pediatric Research, Indiana University School of Medicine, Indianapolis, IN USA; 4 Richard L. Roudebush VA Medical Center, Indianapolis, IN USA; 5 Department of Pediatrics and Diabetes Center, University of California San Francisco, San Francisco, CA USA; 6 CNR Institute of Neurosciences, Padua, Italy; 7 CNR Institute of Clinical Physiology, Pisa, Italy

**Keywords:** Beta cell glucose sensitivity, Clinical trial outcomes, C-peptide AUC, Stage 3 type 1 diabetes, Type 1 diabetes

## Abstract

**Aims/hypothesis:**

At present, no disease-modifying therapies are available for individuals who have been newly diagnosed with type 1 diabetes, despite promising results from decades of clinical trials initiated at stage 3 disease onset. Historically, clinical trials have used changes in the C-peptide AUC (AUC_Cp_) during a mixed meal tolerance test (MMTT) as the primary endpoint; however, this measure does not always correlate with clinical outcomes.

**Methods:**

We analysed 4930 MMTT data points from 799 participants in nine Phase II stage 3 type 1 diabetes trials to determine whether a model-derived physiological measure of in vivo beta cell glucose sensitivity (βGS) could augment clinical trial strategies in type 1 diabetes.

**Results:**

Older age and higher BMI were associated with maintenance of βGS (defined as loss <10% of the baseline value) and maintenance of HbA_1c_ <53 mmol/mol (7.0%). Baseline βGS, age, HbA_1c_ and insulin dose together predicted the magnitude of the effect on HbA_1c_ following intervention. When positive and negative trials were compared, normalised βGS served as an earlier indicator of trial efficacy compared with AUC_Cp_.

**Conclusions/interpretation:**

Our results identified thresholds of change in βGS associated with a clinically significant impact on glycaemic management after intervention and suggest that baseline βGS in association with clinical and demographic parameters may be applied to identify individuals who are more likely to respond to an intervention.

**Graphical Abstract:**

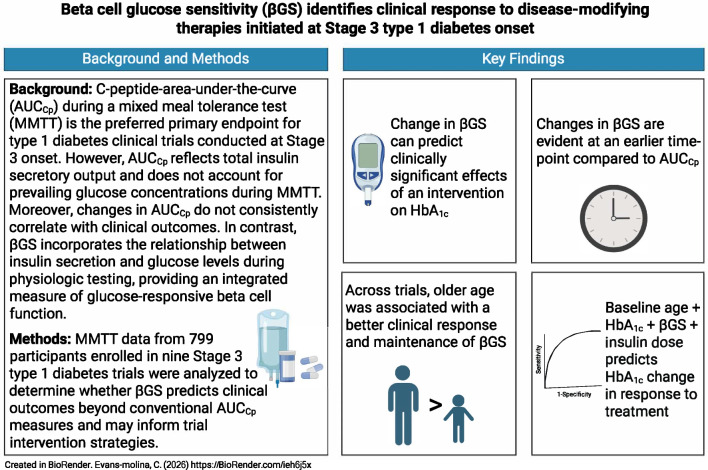

**Supplementary Information:**

The online version contains peer-reviewed but unedited supplementary material available at 10.1007/s00125-026-06703-8.


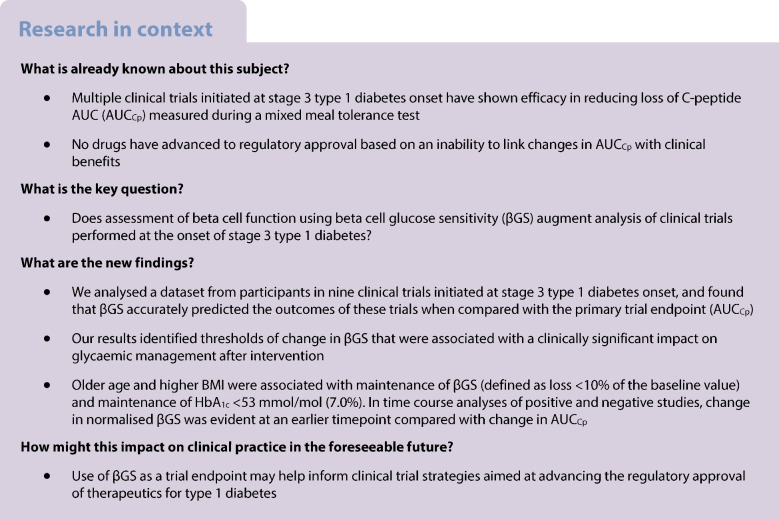


## Introduction

Type 1 diabetes results from immune-mediated destruction of insulin-producing beta cells, leading to chronic hyperglycaemia, lifelong dependence on exogenous insulin therapy, and reduced life expectancy [1, 2]. While the approval of teplizumab to delay the progression from stage 2 to stage 3 type 1 diabetes has changed the landscape of interventions in early-stage disease, clinical trials performed over the past three decades have more commonly focused on interventions in new-onset stage 3 type 1 diabetes. To date, 11 agents administered at this timepoint in Phase II studies have shown efficacy in reducing C-peptide loss 1–2 years after diagnosis [3–5]. However, none of these agents has advanced to regulatory approval, leaving no treatment options aside from insulin replacement for the nearly half a million individuals newly diagnosed with type 1 diabetes each year [6].

Data from observational studies including the DCCT/EDIC study and the Scottish Diabetes Research Network Type 1 Biorepository suggest that the persistence of C-peptide in long-duration disease is associated with improved glycaemic management, reduced frequency of complications, and protection against hypoglycaemia [3, 7–9], all of which are endpoints that are considered clinically meaningful by regulatory agencies. However, linking early changes in C-peptide levels with clinical benefits in long-duration disease has been challenging.

Historically, trials performed at stage 3 type 1 diabetes have used the change in the C-peptide AUC (AUC_Cp_) in response to a standardised mixed meal tolerance test (MMTT) as their primary endpoint [10]. While this measure has been widely adopted by clinical trial networks, it provides incomplete information about beta cell function because it fails to account for the prevailing glycaemia, which per se stimulates insulin release. Moreover, changes in AUC_Cp_ often do not correlate well with clinical markers of metabolic homeostasis, including glycaemic management and exogenous insulin use [11–19]. In addition, rates of decline in AUC_Cp_ after stage 3 diagnosis may vary significantly between trial participants, contributing to heterogeneity in both the magnitude and timing of treatment responses [20, 21]. Therefore, additional measures that more fully describe beta cell function and metabolic homeostasis may help inform efforts to test new agents and advance regulatory approval of therapeutic agents for stage 3 type 1 diabetes.

Previously, we have found that when endogenous insulin secretion during an MMTT or OGTT is analysed relative to the concomitant glucose concentration via a parameter termed beta cell glucose sensitivity (βGS), there is better correlation between beta cell function and clinical measures including HbA_1c_ and exogenous insulin use [22, 23]. Against this background, here we aimed to test whether changes in βGS could be used to inform clinical trial strategies in type 1 diabetes. To this end, we analysed data from nine Phase II recent-onset type 1 diabetes trials [11–19, 24]. Using the entire time course of data from these trials and a pooled placebo group, we examined how serial measurement of βGS changed in response to each immune intervention, and correlated these changes with clinical outcomes. Furthermore, we tested whether changes in βGS could be leveraged as an early indicator of treatment response following an intervention.

## Methods

### Participants

Data from participants in nine Phase II new-onset type 1 diabetes trials were pooled and included in the current study. The aggregated dataset was based on 799 participants, with 533 in the pooled treatment group and 266 in the pooled placebo group (electronic supplementary material [ESM] Table 1). ESM Table 1 also indicates the timing of the primary outcome of each trial based on change in AUC_Cp_ [11–19, 24]. Eligible participants were enrolled within 100 days of diagnosis of stage 3 type 1 diabetes, were positive for at least one diabetes-associated autoantibody (micro-insulin autoantibodies [tested only if the duration of insulin therapy was <10 days], GAD, islet cell antigen 512 [ICA-512], zinc transporter 8 [ZnT8] or islet cell autoantibodies [ICA]), and had a peak stimulated C-peptide of >0.2 nmol/l during an MMTT. Exclusion criteria generally included any serological or clinical evidence of infection, including tuberculosis, hepatitis B or C, or HIV; chronic illness, including cardiac, liver or renal disease; anaemia, leukopenia, thrombocytopenia or neutropenia; ongoing use of diabetes medications other than insulin; vaccination with a live virus within 6 weeks before enrolment; and any other condition that might compromise study participation or confound interpretation of the results. In accordance with SAGER guidelines, self-reported sex was recorded and evaluated in the analysis. Data on gender, ethnicity, regional and socioeconomic factors were not available for this analysis.

All participants had a baseline MMTT at study entry. Thereafter, 758 tests were performed within 6 months from randomisation, 1026 between 6 and 12 months, 726 between 12 and 18 months, 662 between 18 and 24 months, 548 between 24 and 30 months, and 411 between 30 and 68 months. Thus, including the baseline MMTTs (*n*=799), a total of 4930 records were available (1594 for the placebo group and 3336 for the treatments group, i.e. approximately six per participant), with each record corresponding to an individual MMTT.

### Procedures

All participants received intensive diabetes management with the goal of achieving ADA-recommended HbA_1c_ and glycaemic targets. MMTTs were conducted according to standard procedures described in the primary publications [11–19, 24].

### Laboratory tests

Biochemical autoantibodies were assayed at the Barbara Davis Center for Diabetes (Aurora, CO, USA) using radioimmunobinding assays, and islet cell autoantibodies were measured at the University of Florida, as described in the primary publications [11–19, 24]. C-peptide, HbA_1c_ and serum chemistries were measured at the Northwest Lipid Metabolism and Diabetes Research Laboratories (Seattle, WA, USA). All other routine laboratory measures were performed locally.

### Beta cell function model

Beta cell function was evaluated from MMTT glucose and C-peptide concentrations through mathematical modelling [23]. The model describes the relationship between insulin secretion (expressed in pmol min^−1^ m^−2^) and glucose concentration as the sum of two components. The first component represents the dependence of the insulin secretion rate on glucose concentration through a dose–response relationship. From the dose–response relationship, βGS (the mean slope) is calculated. The dose–response relationship is modulated by a potentiation factor, accounting for various mechanisms. The potentiation factor is constrained to a mean of 1 during the test, and reflects the relative potentiation or inhibition of the insulin secretion rate. The excursion of the potentiation factor is quantified by the ratio between the 2 h value and the baseline value (potentiation ratio). The second secretory component represents the dependence of insulin secretion on the rate of change of glucose concentration, and is determined by a single parameter (rate sensitivity), which is related to early insulin release [25]. The model parameters were estimated from glucose and C-peptide concentrations (with use of C-peptide deconvolution [26]), as previously described [23]. Basal insulin secretion rate and total insulin output during the whole test were also calculated. When running the model, cases were flagged if there was an obvious discrepancy between glucose and C-peptide values, suggesting error in measurement. In our analysis, 3.7% of tests were excluded.

### Statistical analysis

Data are presented as means ± SD if the data were normally distributed or as median (IQR) if the distribution was skewed. AUC_Cp_ was computed by the trapezium rule. Quantile plots showing non-normal distribution of βGS, AUC_Cp_ and incremental AUC_Cp_ of the entire cohort are shown in ESM Fig. 1. Group comparisons were performed using the Mann–Whitney U test or the Wilcoxon signed rank test (for unpaired and paired observations, respectively) and the χ^2^ test for categorical variables. Univariate associations were tested by Spearman’s r coefficient. Kaplan–Meier plots were used to compare survival curves by means of the log rank statistic. Cox proportional hazards models were used to estimate HRs and 95% CI. The proportional hazards assumption was confirmed through examination of the log cumulative survival plots. Follow-up durations (i.e. visits with MMTT) were clustered in defined intervals (up to 6, 12, 18, 24, 30 months and study end) equally for each treatment and its placebo.

In these analyses, we applied two endpoints of interest to identify treatment responders. The first endpoint was preservation of beta cell function throughout the duration of follow-up, which we defined as loss of βGS <10% of the baseline value [22]. Of note, the use of ‘10% loss’ as the censor is not the result of a specific threshold analysis, as it is inconsequential to the result: for example, if a censor of ‘50% loss’ instead of ‘10%’ is applied to the rituximab data (HR 0.48; 95% CI 0.42, 0.55; *p*=5.6×10^−28^), the HR is 0.42 (95% CI 0.36, 0.50; *p*=4.5×10^−27^). The only aspect that changes upon raising the level of the censor is the number of events (1269 for ‘10%’, 894 for ‘50%’), i.e. the proportional hazards model finds progressively fewer ‘larger’ changes in βGS. We chose ‘10% loss’ to reflect a relative preservation of beta cell function, in line with other analyses that have defined C-peptide preservation as a minimal loss from baseline [27]. The second endpoint was based on glycaemic management at follow-up, for which we defined a responder as a participant maintaining an HbA_1c_ <53 mmol/mol (7.0%) throughout the duration of follow-up, in accordance with ADA goals for glycaemic management [28]. For the purposes of comparison with the literature, we also used normalised loss AUC_Cp_ (nAUC_Cp_) <10% of the baseline value (in analogy with normalised βGS [nβGS]). Logistic analysis was used to estimate the predictivity of baseline parameters for defined endpoints. *p* values are two-sided, and *p*<0.05 was accepted as statistically significant. For receiver operating curve (ROC) analysis, no adjustment for in-sample optimism was performed. All analyses were performed using JMP version 16.2.0 (SAS Institute, Cary, NC).

## Results

### Study population

The design, methodology and outcome of the individual trials, as reported in the primary publications, are described in ESM Table 1. The participants’ ages ranged from 3.5 to 47 years. The combined anthropometric characteristics and baseline metabolic variables were similar between the pooled placebo arms and the intervention arms. However, participants in trials that met their prespecified endpoint had a higher median age and BMI compared with the trials not meeting their prespecified endpoint (ESM Table 2).

### Normalised βGS predicts trial outcomes and clinical response

**Fig. 1:**
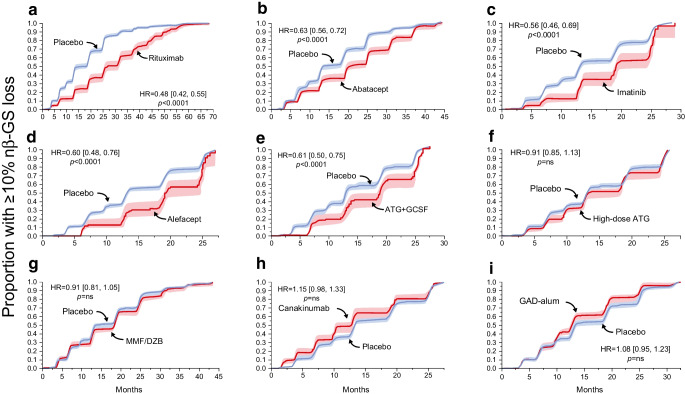
Maintenance of βGS predicts trial outcomes. Kaplan–Meier plots of individual drugs vs pooled placebo event rates showing the proportion of participants with loss of nβGS ≥10% of the baseline value over the entire time period of observation. 95% CI are shaded

Using all available records for each treatment group and loss of nβGS as the endpoint, rituximab, abatacept, imatinib, alefacept and anti-thymocyte globulin (ATG) + PEGylated granulocyte colony-stimulating factor (GCSF) resulted in an overall HR that was significantly less than 1 against placebo based on the Kaplan–Meier estimator, while high-dose ATG, mycophenolate mofetil/daclizumab (MMF/DZB), canakinumab and glutamic acid decarboxylase (GAD)-alum did not (Fig. 1). These results were broadly concordant with the results of the primary trials, which used AUC_Cp_ as the endpoint and one-sided* p* values. The rituximab, GAD-alum, canakinumab, imatinib, ATG/GCSF and high-dose ATG trials each reported their primary outcome at 1 year, while the MMF/DZB, abatacept and alefacept trials reported their primary outcome at 2 years (ESM Table 1).

**Fig. 2:**
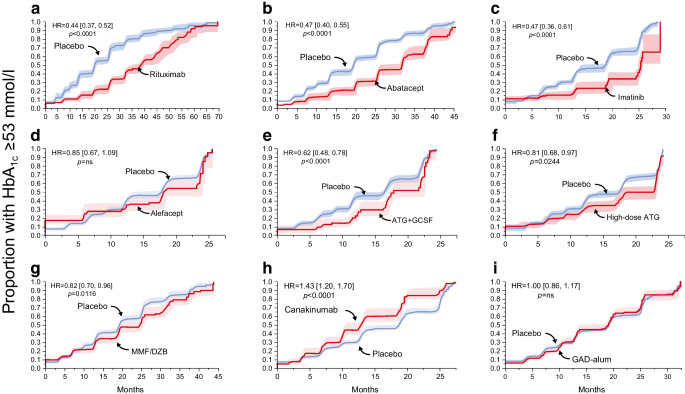
Clinical response to interventions is observed using an aggregated placebo group. Kaplan–Meier plots of individual drugs vs pooled placebo event rates using the proportion of participants exceeding an HbA_1c_ ≥53 mmol/mol (7%) after the baseline visit over the entire time period of observation. 95% CI are shaded

Next we tested the endpoint of maintenance of HbA_1c_ <53 mmol/mol (7.0%). The results were similar to those obtained using nβGS; however, the results for alefacept did not reach statistical significance using this outcome, while those for ATG/GCSF did. The performance of canakinumab was significantly worse than that of the placebo (Fig. 2).

### Demographic features that influence βGS and HbA_1c_

We evaluated demographic features that influenced either the preservation of βGS or nAUC_Cp_ (defined in both cases as loss <10% of the baseline value) or maintenance of HbA_1c_ at <53 mmol/mol (7.0%). Table 1, columns 2–4, shows the HRs for the effect of sex, age and BMI on the βGS endpoint. Using 24 months as the time to endpoint for all trials, multivariate Cox analyses showed that sex was not a significant predictor of response in any trial. Age was a significant predictor in all nine trials, and higher age translated into an approximately 30% relative risk reduction for each 10 year difference. BMI was an independent predictor in all but two trials (rituximab and MMF/DZB). The HRs for the effect of sex, age and BMI on HbA_1c_ and nAUC_Cp_ as endpoints were similar to those observed for βGS but are not shown due to space constraints.

**Table 1:** Results of analysis using multivariate Cox models

Treatment^a^	Sex^b^	Age/10 years^c^	BMI per SD	nβGS ≥10%	HbA_1c_ ≥53 mmol/l (7.0%)	nAUC_Cp_ ≥10%
Rituximab (*n*=267)	1.00 (0.88, 1.14)	0.73 (0.67, 0.80)*	0.94 (0.88, 1.01)	0.74 (0.62, 0.88)*	0.71 (0.56, 0.90)*	0.74 (0.62, 0.88)*
Abatacept (*n*=393)	1.03 (0.91, 1.17)	0.72 (0.66, 0.80)*	0.92 (0.86, 0.98)*	0.74 (0.64, 0.85)*	0.45 (0.37, 0.56)*	0.72 (0.63, 0.84)*
GAD-alum (*n*=552)	0.97 (0.86, 1.09)	0.73 (0.67, 0.80)*	0.92 (0.86, 0.98)*	1.21 (1.06, 1.37)*	0.85 (0.72, 0.99)*	1.23 (1.08, 1.40)*
MMF/DZB (*n*=415)	0.98 (0.87, 1.11)	0.77 (0.71, 0.84)*	0.93 (0.99, 1.08)	1.04 (0.91, 1.19)	0.98 (0.82, 1.16)	1.04 (0.90, 1.19)
Canakinumab (*n*=313)	0.99 (0.88, 1.13)	0.71 (0.65, 0.79)*	0.92 (0.86, 0.98)*	0.93 (0.79, 1.09)	0.98 (0.81, 1.18)	0.92 (0.78, 1.09)
Imatinib (*n*=228)	0.94 (0.82, 1.08)	0.73 (0.66, 0.80)*	0.91 (0.85, 0.98)*	0.81 (0.64, 1.02)	1.12 (0.82, 1.52)	1.10 (0.90, 1.37)
Alefacept (*n*=138)	1.00 (0.87, 1.15)	0.70 (0.66, 0.80)*	0.73 (0.66, 0.80)*	0.71 (0.56, 0.89)*	1.03 (0.79, 1.03)	0.60 (0.45, 0.77)*
ATG/GCSF (*n*=418)	0.97 (0.85, 1.11)	0.72 (0.66, 0.80)*	0.93 (0.86, 1.00)*	0.68 (0.55, 0.84)*	0.73 (0.56, 0.96)*	0.65 (0.52, 0.81)*
High-dose ATG (*n*=155)	0.96 (0.85, 1.08)	0.72 (0.66, 0.79)*	0.92 (0.86, 0.99)*	1.07 (0.92, 1.24)	0.87 (0.72, 1.05)	1.09 (0.94, 1.27)

Values are HR (95% CI)

The numbers in Table 1 indicate records evaluated up to 24 months

Endpoints are nβGS ≥10% loss at any time during 2 years of follow-up after baseline, HbA_1c_ ≥53 mmol/mol (7.0%) at any time during 2 years of follow-up after baseline, or loss of nAUC_Cp_ ≥10% at any time during 2 years of follow-up after baseline. Endpoints in columns 5-7 are adjusted for sex, age and BMI; HRs for sex, age, and BMI are given only for the endpoint of nβGS (columns 2, 3 and 4) for reasons of space, but the results were similar for nAUC_Cp_ and HbA_1c_

^a^The number of records during 24 months of follow-up for each trial is given in parentheses

^b^Male vs female

^c^Age in increments of 10 years

^*^Statistically significant HRs

Next, we analysed the effect of each intervention on loss of βGS (column 5), HbA_1c_ (column 6) or nAUC_Cp_ (column 7) with adjustment for age, sex and BMI. We found a significant beneficial effect of rituximab, abatacept, alefacept and ATG-GCSF on nβGS. Notably, imatinib failed to reach full statistical significance for the nβGS outcome, while GAD-alum showed a negative effect on βGS. Using HbA_1c_ as the endpoint with the same covariates (sex, age and BMI) yielded different results from nβGS for alefacept, which showed no impact on HbA_1c_, and for GAD-alum, which exhibited a positive effect on HbA_1c_. The results for nAUC_Cp_ endpoint were fully concordant with those for nβGS.

When the participants were broken down by age into children (3.5–11.9 years, *n*=173), adolescents (12.0–17.9 years, *n*=326) and adults (≥18 years, *n*=300), a graded increase in loss of beta cell function (nβGS) was apparent over the follow-up period regardless of assigned treatment (ESM Fig. 2).

### Normalised βGS serves as a reliable early indicator of trial efficacy

To examine the time course of the effect of treatments, the adjusted Cox models were run for different times to endpoint. Marked differences emerged among the treatments. For example, the efficacy of imatinib was rapid and concomitant with active treatment but diminished shortly thereafter, whereas the pharmacological effect of rituximab (which was administered for only the initial 4 weeks in the study) was delayed but lasted until the study end (ESM Fig. 3). nβGS predicted a positive outcome at 3 months for imatinib and HbA_1c_ did so for abatacept, but nAUC_Cp_ did not predict a positive outcome for any treatment (Table 2).

**Table 2:** Time course of the results of analysis using multivariate Cox models

Treatment	nβGS ≥10% at 3 months	nβGS ≥10% at 6 months	nβGS ≥10% at 12 months	nβGS ≥10% at 24 months	HbA_1c_ ≥53mmol/l (7.0%) at 3 months	nAUC_Cp_ ≥10% at 3 months
Rituximab	0.98 (0.56, 1.69) [103, 511]	0.86 (0.62, 1.19) [157, 774]	0.75 (0.59, 0.96) [207, 1087]*	0.74 (0.62, 0.88) [301, 1468]*	0.94 (0.62, 1.40) [99, 473]	1.11 (0.63, 1.84) [102, 511]
Abatacept	0.95 (0.64, 1.40) [152, 511]	0.69 (0.54, 0.88) [230, 774]*	0.79 (0.65, 0.95) [302, 1087]*	0.74 (0.64, 0.85) [335, 1468]*	0.52 (0.35, 0.78) [137, 473]*	1.01 (0.68, 1.48) [152, 511]
Gad-Alum	1.06 (0.76, 1.49) [192, 511]	0.86 (0.70, 1.07) [295, 774]	1.02 (0.87, 1.19) [453, 1087]	1.21 (1.06, 1.37) [567, 1468]*	0.78 (0.58, 1.06) [187, 473]	1.18 (0.84, 1.66) [192, 511]
MMF/DZB	0.77 (0.54, 1.10) [163, 511]	1.22 (0.97, 1.53) [243, 774]	1.10 (0.92, 1.31) [317, 1087]	1.04 (0.91, 1.19) [440, 1468]	0.97 (0.72, 1.31) [144, 473]	0.74 (0.51, 1.05) [163, 511]
Canakinumab	1.38 (0.96, 1.97) [138, 511]	1.48 (1.14, 1.92) [183, 774]*	1.13 (0.93, 1.37) [263, 1087]	0.93 (0.79, 1.09) [332, 1468]	0.97 (0.69, 1.40) [92,473]	1.09 (0.72, 1.60) [138, 511]
Imatinib	0.43 (0.20, 0.90) [88, 511]*	0.61 (0.38, 0.98) [121, 774]*	0.80 (0.58, 1.10) [173, 1087]	0.81 (0.64, 1.02) [252, 1468]	1.04 (0.65, 1.65) [87, 473]	1.05 (0.60, 1.78) [88, 511]
Alefacept	nd	1.10 (0.64, 1.90) [64, 774]	0.66 (0.46, 0.95) [93, 1087]*	0.71 (0.56, 0.89) [152, 1468]*	3.45 (2.20, 5.41) [33, 473]*	nd
ATG+GCSF	nd	0.63 (0.41, 0.96) [75, 774]*	0.67 (0.50, 0.90) [111, 1087]*	0.68 (0.55, 0.84) [177, 1468]*	1.41 (0.73, 2.70) [34, 473]	nd
High-dose ATG	1.10 (0.72, 1.69) [114, 511]	0.69 (0.53, 0.91) [185, 774]*	1.06 (0.88, 1.28) [280, 1087]	1.07 (0.92, 1.24) [388, 1468]	1.21 (0.88, 1.68) [114, 473]	1.07 (0.67, 1.66) [114, 511]

Values are HR (95% CI) adjusted for sex, age and BMI; the number of records is given in square brackets (treatment, placebo)

Endpoints are nβGS ≥10% loss within the stated time of follow-up (columns 2–5) or HbA_1c_ ≥53 mmol/mol (7.0%) (column 6) and nAUC_Cp_ ≥10% (column 7) within 3 months of follow-up

^*^Statistically significant HRs

nd, no data

**Fig. 3:**
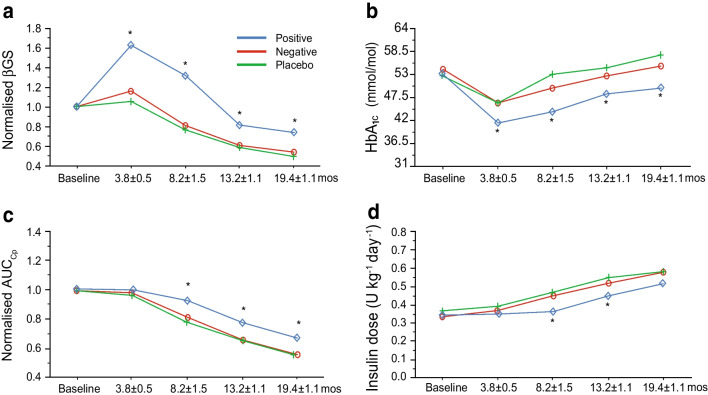
Normalised βGS serves as reliable early indicator of trial efficacy. Time course of (**a**) nβGS, (**b**) HbA_1c_, (**c**) nAUC_Cp_ and (**d**) insulin dose in the four positive vs the three negative studies for which data were available. Note that 3-month data were unavailable for high-dose ATG and alefacept trials. Times shown on the *x* axis are in months (mos). Asterisks indicate statistically significant differences (*p≤0.05) between the positive and negative groups at the indicated time point

Building on this time-to-endpoint analysis, we assessed whether nβGS might serve as an early indicator of trial efficacy. For this analysis, the time course of nβGS was compared with nAUC_Cp_, HbA_1c_ and insulin dose for all trials according to their primary outcome (i.e. whether the original trial was determined to have a positive or negative outcome on C-peptide preservation; ESM Table 1). At the 3-month timepoint, the change in nβGS in drug-treated individuals from positive trials was significantly different when compared with that for drug-treated individuals from negative trials (Fig. 3a). Separation between the groups began at 3 months and continued throughout the observation period. In contrast, when the time course of nAUC_Cp_ was analysed, these groups were not different until around 6 months (Fig. 3c). Individual responses for both nβGS and nAUC_Cp_ in positive trials that included a 3-month timepoint are shown in ESM Fig. 4. The time course of HbA_1c_ showed a similar pattern to that observed with nβGS (Fig. 3b). The insulin dose increased in both groups, with a 6-month delay in the positive vs the negative studies (Fig. 3d).

### Baseline age, HbA_1c_, insulin dose, and βGS efficiently predict changes in HbA_1c_ at 1 year

**Fig. 4:**
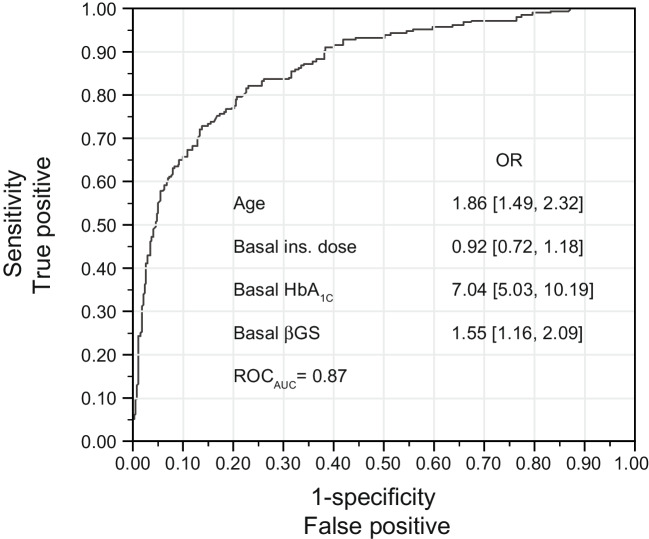
Prediction of the proportion of participants achieving a decrease in HbA_1c_ of ≥5.5 mmol/mol (0.5%). Multivariate logistic model for the proportion of participants achieving a decrease in HbA_1c_ of ≥5.5 mmol/mol (0.5%) from baseline in the entire database using four baseline parameters and ROC analysis. The ORs are multivariate and correspond to one SD increment of each variable. Glucose sensitivity is log-transformed. The corresponding univariate ORs are 1.65 (95% CI 1.41, 1.94) for age, 1.01 (95% CI 0.85, 1.20) for insulin dose, 5.14 (95% CI 3.98, 6.79) for HbA_1c_, and 0.91 (95% CI 0.77, 1.08) for βGS. No adjustment for in-sample optimism was performed in the ROC analysis

Finally, we sought to determine whether baseline (pre-randomisation) parameters could predict changes in HbA_1c_ at 1 year of follow-up. We found that a linear combination of age, HbA_1c_, insulin dose and βGS efficiently predicted (with a ROC_AUC_ of 0.87) the proportion of participants with a decrease in HbA_1c_ of at least 5.5 mmol/mol (0.5% at 1 year) (Fig. 4).

## Discussion

Despite over 30 years of clinical trial efforts, there remains a gap in translating clinical trial results from Phase II stage 3 type 1 diabetes interventions into registration trials and subsequent clinical practice. The reasons for this gap are multifactorial, but importantly include debate surrounding the relationship between C-peptide and direct clinical benefits. The integrated measure of C-peptide response during an MMTT has been used extensively in natural history and intervention studies as a proxy of beta cell function. However, changes in the C-peptide response (AUC_Cp_) are heterogeneous amongst adults and children, necessitating trials of long duration, and often did not correlate well with clinical measures of benefit (i.e. glycaemic management, glycaemic variability, protection against hypoglycaemia, and exogenous insulin use) in individual trials [3, 5, 20, 27, 29].

Here, we analysed participant-level data from nine Phase II clinical trials of immune interventions in participants with new-onset type 1 diabetes (stage 3) using serial measurements of beta cell function modelled by the measure βGS [11–19, 24]. In contrast to AUC_Cp_, βGS incorporates the relationship between insulin release and plasma glucose concentrations during physiological testing, thus providing a more direct and accurate assessment of in vivo beta cell function. Across these trials, our results identified thresholds of change in βGS associated with a clinically significant impact on glycaemic management after intervention. In addition, we found that baseline βGS, especially in association with age, HbA_1c_ and insulin dose, can predict the magnitude of effect on HbA_1c_, and that this relationship can be easily modelled in pre-trial design strategies. In addition, by comparing positive and negative trials, we found that, in contrast to changes in AUC_Cp_, increases in βGS are evident early in the treatment course (i.e. at 3 months) in positive studies, implying that beta cell dysfunction can be in part improved by immune intervention. Finally, by comparing the time course of response in the various trials, we found that βGS analysis can provide important insights into pharmacodynamic responses to specific drugs. Taken together, these findings suggest that βGS can help inform stage 3 type 1 diabetes clinical trial efforts.

Recent studies have examined aggregated data from stage 3 clinical trials, with several key themes emerging. In the TOMI meta-analysis of 21 trials, aggregate AUC_Cp_ data from positive and negative trial results showed that approximately 76% preservation of AUC_Cp_ was sufficient to maintain glycaemic control [29]. The authors concluded that the failure of individual studies to link improvements in AUC_Cp_ with clinical benefit may be partly due to inadequate statistical power. Indeed, one challenge in designing type 1 diabetes studies and interpreting the results has been the wide heterogeneity in the change in beta cell function over time in individuals with type 1 diabetes [21]. As age is an important determinant of heterogeneity, Ylescupidez et al [30] used a quantitative response metric incorporating age and AUC_Cp_, and showed that this heterogeneity applies also to differences between placebo groups in various trials, which may have additional implications for trial interpretation.

Here, we aimed to expand on these concepts. Similar to the TOMI and Ylescupidez analyses, we used an aggregated placebo group. However, we applied a more physiological measure of beta cell function that incorporates changes in glycaemia, and our analysis leveraged the complete time course of MMTT measures. The beta cell function model employed was originally developed and applied in cohorts of individuals with normal or impaired glucose tolerance and type 2 diabetes [31–33]. Previously, we have used this model to analyse the longitudinal natural history of beta cell function decline before and after stage 3 type 1 diabetes onset to show that βGS is predictive of progression to type 1 diabetes in autoantibody-positive individuals and that alterations in βGS precede changes in glucose levels, insulin secretion or insulin sensitivity based on OGTT assessments. In placebo-treated trial participants with new-onset stage 3 type 1 diabetes, baseline βGS was predictive of glycaemic control 2 years after diagnosis, highlighting the potential of this measure to identify clinically meaningful outcomes in type 1 diabetes interventions [22, 34, 35].

In this analysis, we compared data from the placebo and treatment arms of nine trials, and found that outcomes based on βGS broadly agreed with outcomes based on a change in AUC_Cp_, when applying a definition of response as a loss of less than 10% of the baseline value of either βGS or AUC_Cp_. In addition, applying a glycaemic endpoint during 24 months of follow-up, we could contrast changes in βGS with maintenance of an HbA_1c_ <53 mmol/mol (7.0%). Of the trials analysed, those for rituximab, abatacept and ATG/GCSF had concordant beneficial effects on βGS and HbA_1c_. A notable exception was observed for canakinumab, which worsened glycaemic control even though the βGS was no different between treatment- and placebo-treated individuals.

We used multivariate Cox models to explore factors associated with maintenance of βGS and HbA_1c_. Sex did not drive apparent differences in response. In contrast, we found that older age was associated with better clinical outcome and maintenance of βGS. Intriguingly, Taylor et al found also that the combined median age in the analysed positive trials was slightly but significantly higher compared with the negative trials that did not meet their prespecified endpoint [29]. Using the full database, we confirmed that adults exhibit a slower rate of decline in βGS after stage 3 onset compared with children and adolescents [22]. This slower rate of decline and possible inability to show a large effect size for an intervention has the potential to discourage adult inclusion in trials [36]. However, the analysis in the present study indicates that adults may derive significant benefit from interventions, and suggests that new-onset studies in this population, with consideration of alternative endpoints that provide a more granular assessment of beta cell function, are warranted. Our analysis showed also that baseline βGS was a strong predictor of clinical response during 12 months of follow-up, which is similar to observations made using AUC_Cp_ [29]. Moreover, we found that a combination of age, HbA_1c_, insulin dose and βGS could be used to predict a clinically significant 0.5% decrease in HbA_1c_ at 1 year [37].

Finally, when the adjusted Cox model was evaluated for different times to endpoint, marked differences in βGS dynamics in response to various therapeutics were apparent. The most extreme differences were noted when comparing the impact of imatinib and rituximab on βGS. When trial participants were treated with imatinib, there was a rapid improvement and stabilisation of βGS that was lost after active treatment ceased. In contrast, treatment with rituximab showed a delayed impact on βGS that lasted until 24 months. Thus, βGS analysis offers further insights into the pharmacodynamics of different drugs, and may be helpful in guiding future use of therapeutic agents or combination approaches. When comparing successful therapies and those that failed to reach their primary endpoint (based upon AUC_Cp_), it is notable that using βGS as the endpoint amplifies these conclusions; βGS improves significantly in the initial 3 months in successful studies, closely mirroring changes in HbA_1c_. These early outcome differences were much less noticeable when using AUC_Cp_ as the endpoint. βGS analysis may help to identify early responses to therapies, and its use could support adaptive trial designs or cessation of trials that are exhibiting futility, especially when combined with clinical measures such as HbA_1c_.

The strengths of this study include the large group of participants, drawn from nine Phase II type 1 diabetes clinical trials performed close to stage 3 onset. The trials represent a wide variety of different therapeutic approaches, and included five successful and four unsuccessful trials based on the prespecified primary endpoint. Participants were well characterised through serial assessments over a 2-year period, which greatly increased the statistical power of the analysis. However, there are limitations with any post hoc analyses of clinical trial data that must be acknowledged. Prospective application of βGS will need to be studied and validated in future trials. In addition, the participants were largely of European descent and non-Hispanic, and the <12 year age group was somewhat under-represented compared with the older age groups. An additional weakness of our analysis is that individuals who agree to participate in clinical research trials may not be representative of a standard clinical population. They may have been more carefully monitored and had more intensive diabetes management by virtue of study participation, which could impact the application of clinical endpoints. We analysed trials that enrolled participants within 100 days of diagnosis. It is not clear whether the results of our analysis would differ if individuals were enrolled closer to diagnosis. Finally, there is no standard or accepted definition of a trial responder in type 1 diabetes clinical trials. Here, we defined response as loss <10% of the baseline value for βGS or maintenance of HbA_1c_ <53 mmol/mol (7.0%), whereas other groups have defined response using a variety of metrics, sometimes combining metabolic and immunological endpoints [38–42].

Notwithstanding these limitations, we found that βGS offers an additional means to assess change in beta cell function over time for those participating in a stage 3 intervention. These data add to the growing body of literature focused on aggregate analysis of stage 3 trials, and highlight bona fide clinical benefits of disease-modifying interventions on beta cell function at this stage. In aggregate, the results of our study and recent other studies suggest that alternative measures of beta cell function (in combination with clinical features), as well as the use of historical aggregated control data and ‘synthetic’ placebo groups, could help to accelerate type 1 diabetes clinical trial efforts.

## Supplementary Information

Below is the link to the electronic supplementary material.

ESM (PDF 561 KB)

## Data Availability

TrialNet clinical trials data are publicly available, and can be obtained by application to the NIDDK Central Repository at https://repository.niddk.nih.gov/home/. Immune Tolerance Network clinical trials data are also publicly available at https://www.itntrialshare.org/. Data from the imatinib study are available from SEG per data sharing statements from the original publication [24]. Requests for any additional data may be made by contacting the corresponding author.

## Abbreviations

ATG: Anti-thymocyte globulin
AUC_Cp_: C-peptide AUC
βGS: Beta cell glucose sensitivity
DZB: Daclizumab
GAD: Glutamic acid decarboxylase
GCSF: PEGylated granulocyte colony-stimulating factor
MMF: Mycophenolate mofetil
MMTT: Mixed meal tolerance test
nβGS: Normalised beta cell glucose sensitivity
nAUC_Cp_: Normalised C-peptide AUC
ROC: Receiver operating curve

## Authors’ relationships and activities

CE-M has served on advisory boards related to type 1 diabetes research clinical trial initiatives: Isla Technologies, Neurodon and DiogenX. CE-M receives in-kind research support from Bristol Myers Squibb and has received investigator-initiated grants from Lilly Pharmaceuticals and Astellas Pharmaceuticals. CE-M owns a patent (16/291,668): ‘Extracellular Vesicle Ribonucleic Acid (RNA) Cargo as a Biomarker of Hyperglycaemia and Type 1 Diabetes’, and a provisional patent (63/285,765): ‘Biomarker for Type 1 Diabetes (PDIA1 as a biomarker of beta cell stress)’. SEG has served on advisory boards for Abata Therapeutics, Genentech, GentiBio, Provention Bio, SAB Biotherapeutics, Sana Biotechnology, Sanofi and Shoreline Biosciences, has received support from the NIH, Provention Bio and Sanofi for roles as an investigator in clinical trials, and serves on data and safety monitoring boards for Diamyd Medical, Breakthrough T1D and INNODIA. AM is a consultant for Eli Lilly & Co. and Novo Nordisk (on GIP/GLP-1RAs). EF is a member of the Scientific Advisory Board for Eli Lilly & Co. and Oramed, has given talks at Eli Lilly & Co. and Boehringer Ingelheim, and receives research grant support from Janssen, Oramed and the NIDDK.
