# Supplementary material for: Beta cell glucose sensitivity identifies clinical response to disease-modifying therapies initiated at stage 3 type 1 diabetes onset
Source: Diabetologia. 2026 Mar 12;69(6):1482–93. doi: 10.1007/s00125-026-06703-8 (PMC13109176; doi:10.1007/s00125-026-06703-8)
Supplement: Supplementary file 1 — ESM (PDF 561 KB) [file 125_2026_6703_MOESM1_ESM.pdf]

Electronic Supplementary Material

Beta cell glucose sensitivity identifies clinical response to disease-modifying therapies initiated at Stage 3 type 1 diabetes onset

Carmella Evans-Molina, Stephen E. Gitelman, Andrea Mari, and Ele Ferrannini

ESM Table 1 – Studies analyzed

Agent Tested	Mechanism of Action	Placebo (n)	Treatment (n)	Reported Endpoint
**Rituximab**[a,b]	Anti-CD20 MAB	29	55	2 hr AUC C-peptide at 1 y
**Abatacept**[a,b]	Co-stimulatory modulator	35	77	2 hr AUC C-peptide at 2 y
**GAD-alum**[b]	Antigen-based immunotherapy	48	97	2 hr AUC C-peptide at 1 y
**Mycophenolate Mofetil & Daclizumab**[b]	Anti-lymphocyte; anti-CD25 MAB	42	84	2 hr AUC C-peptide at 2 y
**Canakinumab**	Anti-IL-1beta MAB	22	47	2 hr AUC C-peptide at 1 y
**Imatinib**[a,b]	Tyrosine kinase inhibitor	22	44	2hr AUC C-peptide at 1 y
**Alefacept**[a,b]	Fusion protein that binds CD2	16	33	2 hr and 4 hr AUC C-peptide at 2 y
**ATG+GCSF**[a,b]	Low-dose ATG	32	58	2 hr AUC C-peptide at 1 y
**High-dose ATG**[b]	High dose ATG	20	38	2 hr AUC C-peptide at 1 y
	Total (n)	266	533	

[a]Trial met pre-specified primary endpoint**.** [b] 4hr MMTT performed at selected timepoints

ESM Table 2 – Clinical and metabolic characteristics of the groups at baseline

	Placebo	Positive[a]	Negative[b]	*p* Pos. *vs* Neg.	*p* Pos. *vs* Plb.	*p* Neg. *vs* Plb.
n	266	247	286	-	-	-
Gender (F/M)	105/161	103/144	127/159	ns	ns	ns
	**Median [IQR]**	**Median [IQR]**	**Median [IQR]**	-	-	-
Age (years)	15.2 [10.6]	17.9 [12.1]	14.6 [7.8]	<0.0001	0.0075	0.0358
BMI ($kg \cdot m^{-2}$)	21.2 [5.8]	22.4 [5.1]	21.2 [5.8]	0.0011	0.0100	ns
HbA_{1c} (%)	6.8 [1.7]	6.8 [1.6]	7.0 [1.7]	ns	ns	ns
Daily insulin dose ($U \cdot kg^{-1}$)	0.32 [0.29]	0.30 [0.30]	0.35 [0.26]	ns	ns	ns
Fasting glucose (mmol/L)	6.1 [1.6]	6.0 [1.4]	6.0 [1.9]	ns	ns	ns
2-hour glucose (mmol/L)	10.6 [4.8]	10.2 [4.8]	10.4 [4.6]	ns	ns	ns
Fasting insulin secretion rate ($pmol \cdot min^{-1} \cdot m^{-2}$)	51 [28]	47 [41]	51 [35]	ns	ns	ns
Total insulin secretion ($nmol \cdot m^{-2}$)	26 [16]	27 [17]	25 [15]	0.0021	ns	ns
Glucose sensitivity ($pmol \cdot min^{-1} \cdot m^{-2} \cdot mM^{-1}$)	1.4 [16.7]	15.5 [15.5]	14.2 [16.4]	ns	ns	ns
Rate sensitivity ($nmol \cdot m^{-2} \cdot mM^{-1}$)	269 [332]	257 [327]	300 [310]	ns	ns	ns
Potentiation ratio	1.18 [0.45]	1.23 [0.52]	1.15 [0.41]	0.0487	ns	ns

[a]Positive studies = rituximab, abatacept, alefacept, imatinib, and ATG/GCSF; [b]Negative studies = high-dose ATG, GAD-alum, MMF/DZB, and canakinumab. IQR = interquartile range; *p* values by Wilcoxon each-pair test.

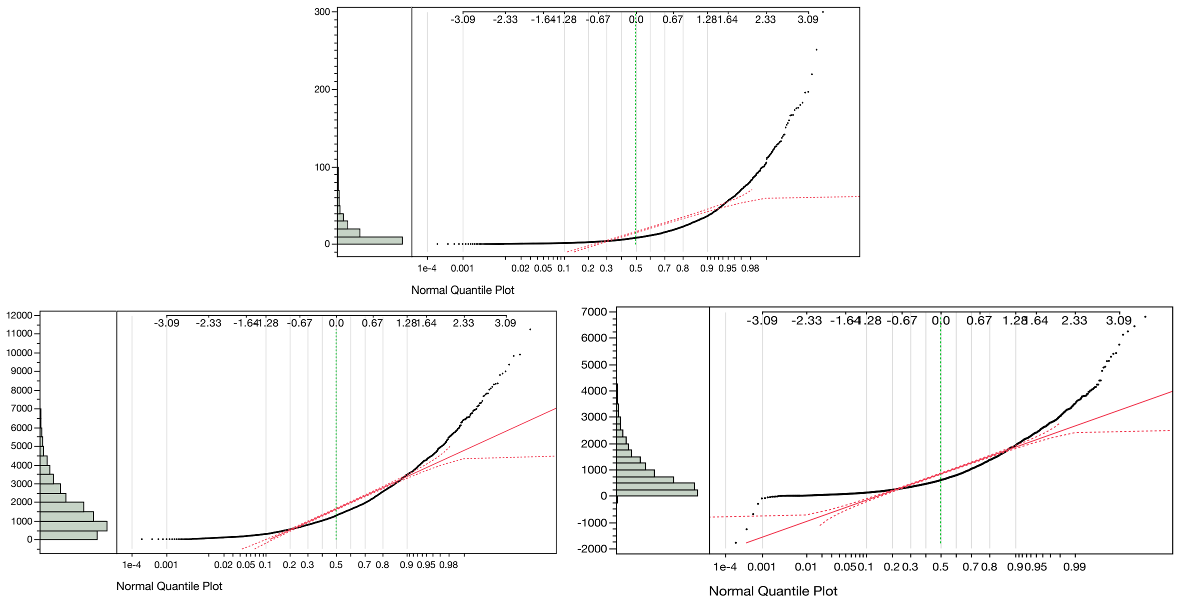

ESM Figure 1 – Quantile plots showing non-normal distribution of beta cell glucose sensitivity, AUC_{Cp}, and incremental AUC_{Cp} across the entire cohort.

Quantile plots showing the non-normal distribution of ßGS (top), AUC_{Cp} (bottom left), and incremental AUC_{Cp} (bottom right). The left plots in the panels show the histogram of the variable.

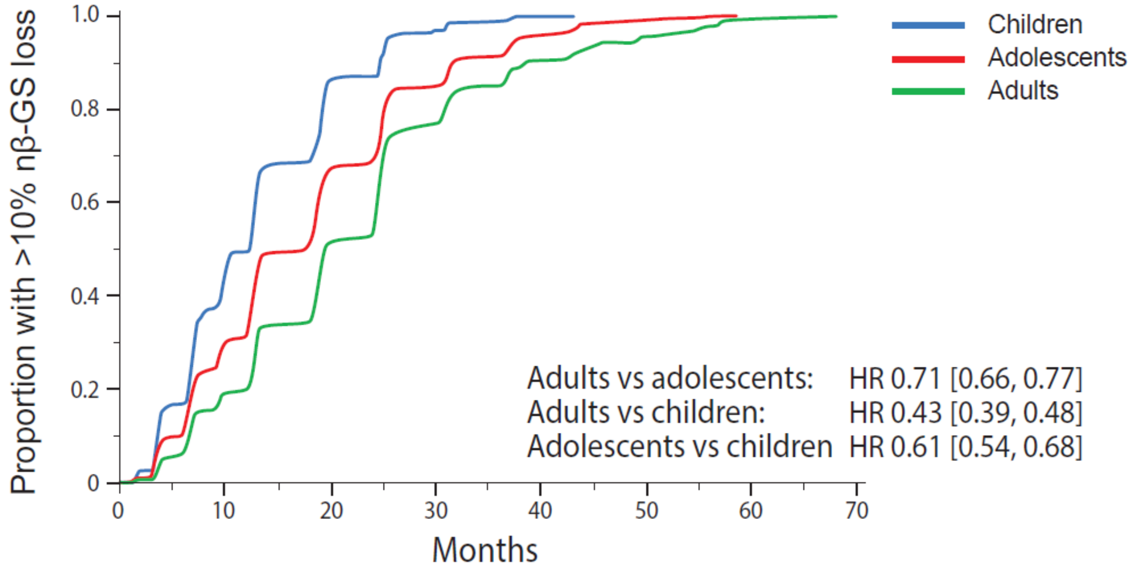

ESM Figure 2 – Impact of defined age brackets on relative risk.

Kaplan-Meier plot of proportion of subjects with >10% loss of normalized beta cell function (nßGS) by age interval. HRs are adjusted by treatment (positive, negative, or placebo).

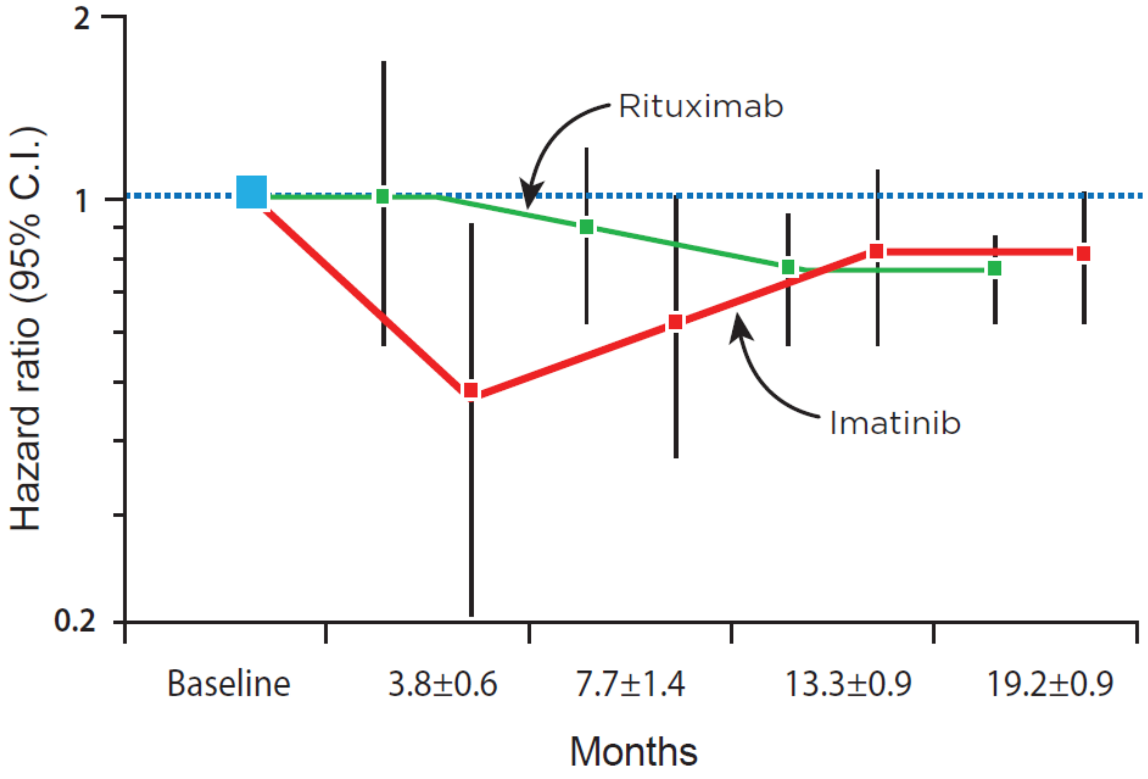

ESM Figure 3 –Representative time-course of change in nßGS in individuals treated with imatinib or rituximab.

Time-course of hazard ratio (and 95% confidence interval) against pooled placebo of normalized beta cell glucose sensitivity (nßGS) contrasting imatinib and rituximab.

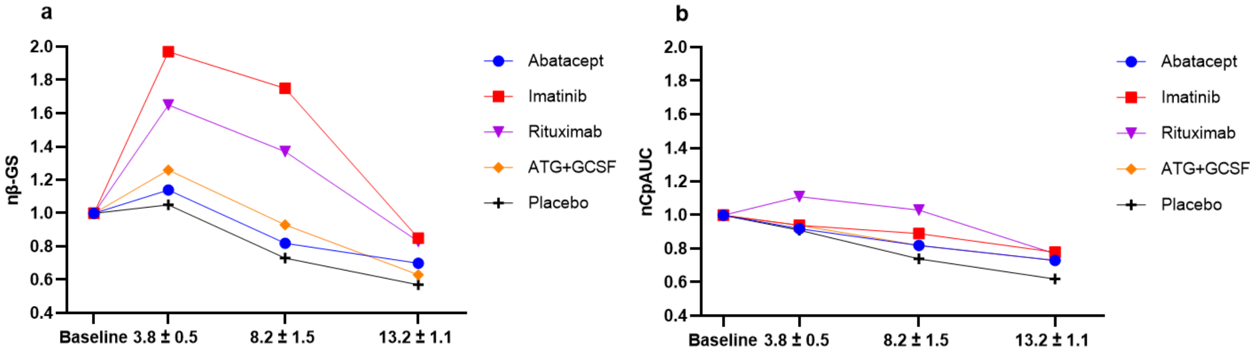

ESM Figure 4 – Individual trial results contributing to Main Figure 3.

Time-course of (**a**) nßGS and (**b**) $nAUC_{Cp}$ for positive studies included in Main Figure 3. Time-course for the combined placebo group is shown in blue.
